# Lavender Essential Oil and Its Terpenic Components Negatively Affect Tumor Properties in a Cell Model of Glioblastoma

**DOI:** 10.3390/molecules29246044

**Published:** 2024-12-22

**Authors:** Miriam Russo, Noemi Martella, Deborah Gargano, Francesca Fantasma, Chiara Marcovecchio, Veronica Russo, Maria Antonietta Oliva, Marco Segatto, Gabriella Saviano, Sabrina Di Bartolomeo, Antonietta Arcella

**Affiliations:** 1 Department of Biosciences and Territory, University of Molise, 86090 Pesche, Italy; m.russo15@studenti.unimol.it (M.R.); n.martella@studenti.unimol.it (N.M.); d.gargano@studenti.unimol.it (D.G.); fantasma@unimol.it (F.F.); c.marcovecchio1@studenti.unimol.it (C.M.); marco.segatto@unimol.it (M.S.); 2 IRCCS Istituto Neurologico Mediterraneo NEUROMED, Via Atinense 18, 86077 Pozzilli, Italy; veronica.2306@hotmail.it (V.R.); mariaantonietta.oliva@neuromed.it (M.A.O.); arcella@neuromed.it (A.A.)

**Keywords:** GBM, essential oils, *Lavandula angustifolia*, terpenes, cell proliferation, cell migration, oxidative stress

## Abstract

Glioblastoma (GBM) is the most common and aggressive form of brain cancer in adults, characterized by extensive growth, a high recurrence rate, and resistance to treatment. Growing research interest is focusing on the biological roles of natural compounds due to their potential beneficial effects on health. Our research aimed to investigate the effects of lavender essential oil (LEO) on a GBM cell model. Chemical characterization using GC-MS analysis indicated that LEO contains several terpenes, compounds that have been found to exhibit anticancer properties by interfering with key cancer-related pathways in several cancer models. By means of cell biology assays, we demonstrated that LEO impairs cell proliferation and migration, and also reduces oxidative stress in U87 cells. We further observed that Terpinen-4-ol, contained in LEO, was capable of reproducing the effects of the oil on GBM cells. Our results suggest that the terpenic molecules present in LEO could be considered valuable allies alongside conventional therapies against GBM.

## 1. Introduction

GBM is the most prevalent and lethal form of brain cancer in adults, with only modest improvements in survival rates over the past thirty years [1,2]. Histologically, GBM is distinguished by its aggressive features: marked mitotic activity, substantial angiogenesis, cellular heterogeneity, necrosis, and rapid proliferation [3,4]. Current treatments for GBM, which include extensive surgical resection followed by radiotherapy and chemotherapy with temozolomide (TMZ), have limited success in preventing tumor progression and infiltration [5,6]. Consequently, the median survival time for GBM patients is approximately 14 months, with most experiencing relapses at various intervals post-treatment [7,8]. In fact, GBM frequently recurs due to its invasive nature and the difficulty of eradicating all cancer cells, including a subpopulation of cancer stem cells. Moreover, the development of TMZ resistance also represents a major obstacle in GBM treatment [9,10]. Considering these limitations, research focuses on unraveling the molecular pathways involved in gliomagenesis and on identifying novel potential therapeutic targets [11,12,13]. Several natural compounds have also been evaluated in recent years for their antitumor properties, including their ability to inhibit tumor growth and invasiveness, and to improve overall patient prognosis in various cancer models [14,15,16]. Some of them have shown significant antitumor activities when combined with TMZ in GBM-resistant cells [17,18].

Lamiaceae plants, including *Lavandula angustifolia*, have traditionally been used to alleviate anxiety, insomnia, and various neurological conditions, as well as to combat infections, manage pain, and address a range of other ailments [19]. In particular, lavender essential oil (LEO) has been shown to be effective in preventing some chemotherapy-induced side effects in human leukemia cells and in xenograft models of human prostate cancer [20,21,22], and in modulating cholesterol metabolism in a cellular model of hepatocarcinoma [23]. LEO’s versatile properties underscore its potential as a natural remedy with diverse health benefits, positioning it as an intriguing topic for scientific research and therapeutic investigation.

LEO contains approximately one hundred biologically active compounds, several of which with considerable therapeutic potential [24]. In particular, terpenes, which are prominent in LEO, have become focal points of biochemical and molecular research due to their wide-ranging biological activities, including anti-inflammatory, antimicrobial, and antiviral properties [25,26]. Terpenes interact with specific biological targets such as enzymes, receptors, and ion channels, thereby influencing crucial signaling pathways involved in apoptosis, proliferation, and cell differentiation [27,28]. In line with this evidence, recent studies indicate that terpenic molecules such as borneol, linalool, and 1,8-cineole present in essential oils can suppress cell cycle progression and induce apoptosis in various cancer cells [29,30,31,32,33]. Among these promising bioactive molecules, terpinen-4-ol, a monocyclic monoterpenoid, exhibits notable antitumor properties by inducing ferroptosis and inhibiting cell proliferation in leukemia and melanoma cells [34,35,36,37]. Additionally, this monoterpene has significant antioxidant effects, demonstrated by its ability to increase the expression of enzymes that support oxidative metabolism homeostasis, both in vivo and in vitro [36]. No evidence is present in the literature concerning the effects of LEO administration on GBM models, although very recently it has been reported that terpinen-4-ol induces the ferroptosis of glioma cells and that borneol enhances the efficacy of TMZ in vitro [32,34]. In this context, our study aimed to investigate the impact of LEO and of its terpenic components on the properties of GBM cells in an in vitro model. We demonstrated that LEO induces a proliferation slowdown and an impairment of cell migration of GBM cells besides reducing oxidative stress. Furthermore, we observed that terpinen-4-ol mimics the anti-oncogenic properties of LEO on GBM cells.

## 2. Results

### 2.1. Extraction and Characterization of L. angustifolia Essential Oil

**Figure 1:**
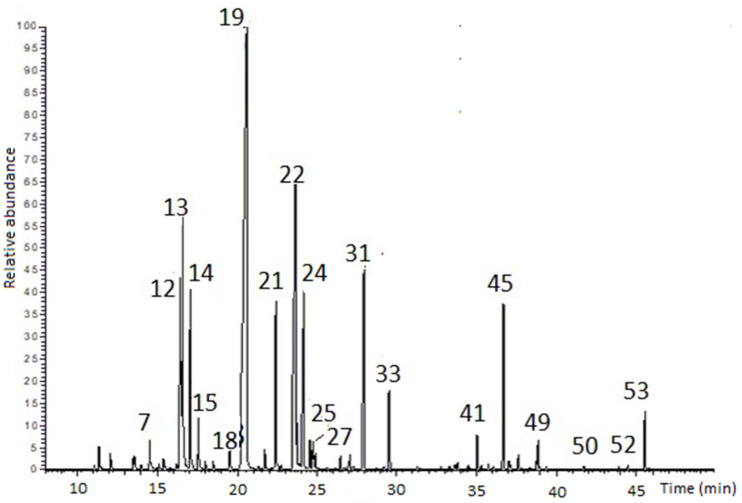
The GC-MS TIC chromatogram of LEO. In the graph, each main component of the oil was labeled with a number (N) based on its elution order, as reported in Table S1.

*L. angustifolia* flowers were collected in Pesche (IS, Southern Italy) during the flowering period. After drying, they were hydrodistillated to obtain an essential oil (LEO) in a yield of 4.5%, calculated according to the dry weight of 100 g. The GC-MS analysis found 53 individual components, corresponding to 98.1% of the total peak areas of the chromatogram (Figure 1). The main chemical components of LEO with an area percentage > 1% are reported in Table 1; all compounds, listed according to their elution order on a Rtx^®^-5 Restek capillary column, are reported in Supplementary Table S1. The complete identification was performed by calculating the experimental retention indices (Exp RI) and comparing them with those found in the literature (Ref RI) [38]. GC-MS analysis confirmed the presence of linalool (33.99%) as the major component and characterizing essence of *L. angustifolia*. The LEO showed high content levels of borneol (13.21%), 1,8-cineole (6.29%), and terpinen-4-ol (5.24%). The oil also contained a mixture of components, mainly oxygenated monoterpenes (73.06%), followed by monoterpenes (15.59%), sesquiterpenes (6.04%), and oxygenated sesquiterpenes (2.81%), as reported in Supplementary Table S2.

### 2.2. LEO Triggers the Dose-Dependent Inhibition of U87MG Cell Proliferation

**Figure 2:**
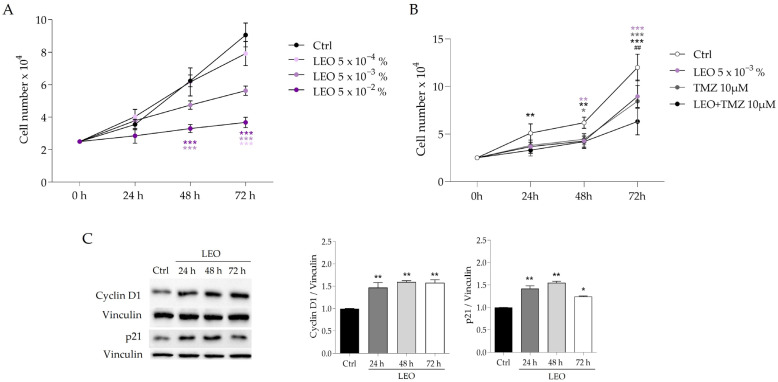
LEO administration reduces the proliferation of U87MG cells. (**A**) U87 cells were cultured in complete DMEM with DMSO as vehicle (Ctrl) or treated with of 5 × 10^−4^, 5 × 10^−3^, and 5 × 10^−2^% (*v*/*v*) of LEO for 24, 48, and 72 h. At the specified time points, the cells were trypsinized and counted in a Blutzählkammer THOMA chamber, and growth curves were plotted. *** *p* < 0.001. (**B**) U87MG cells were cultured in DMEM and treated with 5 × 10^−3^% (*v*/*v*) LEO, 10 μM TMZ and LEO + TMZ for 24, 48, and 72 h. Cells were then trypsinized and counted as in (**A**). Data are presented as means ± SD from three independent experiments. Statistical analysis was performed using the two-way ANOVA and Bonferroni’s post hoc test. Asterisk indicates statistical difference vs. Ctrl group; ## indicates statistical difference vs. TMZ group, *p* < 0.01; * *p* < 0.05, ** *p* < 0.01; *** *p* < 0.001. (**C**) Western blot analysis of p21 and Cyc D1 proteins in U87MG cells treated with 5 × 10^−3^% (*v*/*v*) for 24, 48, and 72 h. Vinculin was used as loading control. Data are presented as means ± SD from three independent experiments. Statistical significance is assessed with one-way ANOVA test, followed by Tukey’s post hoc and indicated vs. Ctrl as follows: * *p* < 0.05; ** *p* < 0.01.

In order to investigate the biological effects of LEO on the U87 GBM cell line, we first evaluated its impact on cell proliferation. Specifically, LEO was administered at concentrations of 5 × 10^−4^, 5 × 10^−3^, and 5 × 10^−2^% in culture medium (*v*/*v*), and the counting of living cells was performed at 24, 48, and 72 h of treatment. We observed a dose-dependent reduction in cell proliferation, which was statistically significant after 48 h and more evident after 72 h, at all doses used (Figure 2A). In detail, 50% inhibition of cell proliferation was observed when cells were grown in the presence of 5 × 10^−3^% (*v*/*v*) LEO for 72 h when compared to control cells treated with the vehicle DMSO (Ctrl) (Figure 2A). Based on the results obtained with the different LEO doses tested, we selected the 5 × 10^−3^% (*v*/*v*) concentration for the subsequent experiments as it proved to be the minimum effective dose in impacting cell proliferation. We also tested the impact of LEO on U87MG treated with TMZ, the first-line chemotherapeutic agent employed for GBM. As shown in Figure 2B, TMZ began to inhibit cell proliferation at 24 h, and the co-administration of LEO and TMZ resulted in a significant reduction in cell proliferation, by over 50% compared to the control after 72 h, thus indicating that LEO enhances the antiproliferative property of TMZ. Consistent with the inhibitory effect on cell proliferation, we also observed an alteration in the expression level of some proteins involved in cell cycle regulation upon LEO treatment. Specifically, the expression of p21, a key cyclin-dependent kinase inhibitor, increased, starting from 24 h of treatment with 5 × 10^−3^% (*v*/*v*) LEO (Figure 2C). Additionally, the expression of cyclin D1, a crucial protein in regulating cell cycle progression, significantly increased from 24 h after treatment with 5 × 10^−3^% (*v*/*v*) LEO, thus suggesting an alteration of the cell cycle machinery induced by LEO (Figure 2C).

### 2.3. LEO Treatment Impairs Migratory Abilities of U87MG Cells

**Figure 3:**
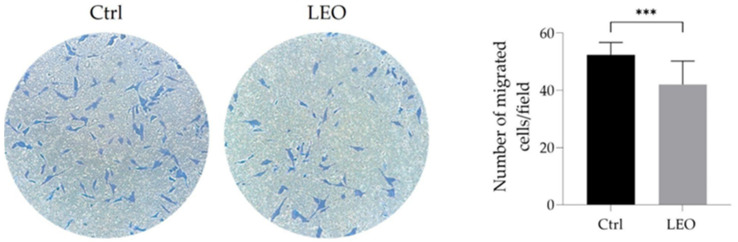
LEO treatment impairs U87MG cell migration. Representative images (20x magnification) of Transwell migration assay of Ctrl and 5 × 10^−3^% (*v*/*v*) LEO-treated U87MG cells and quantitative analysis of the relative number of migrating cells/field. Data are presented as means ± SD from three different experiments. Statistical analysis was performed using the unpaired Student’s *t*-test. *** *p* < 0.001.

The migration ability of cancer cells is a key indicator of their degree of malignancy and is often targeted to contrast cancer aggressiveness or to evaluate anticancer drug efficacy. The ability of LEO to affect the migration of U87MG cells was evaluated by Transwell assays. We observed that a 24 h treatment with LEO markedly diminished the migratory capacity of U87MG cells, as shown in Figure 3. In detail, the presence of 5 × 10^−3^% (*v*/*v*) of LEO led to a significant reduction of the chemotactic response of GBM cells to fetal bovine serum (FBS) stimulus. The impairment of cell chemotaxis highlights the potential inhibitory effect of LEO on the motility of GBM cells, suggesting its interference with cellular mechanisms essential for tumor spreading.

### 2.4. LEO Reduces Oxidative Damage in U87MG Cells

**Figure 4:**
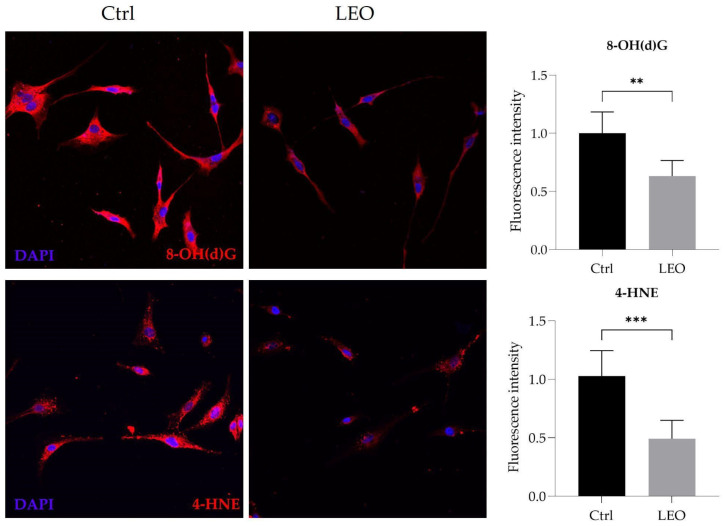
LEO reduces oxidative damage in GBM cells. U87MG cells were cultured in DMEM with DMSO (Ctrl) or treated with 5 × 10^−3^% (*v*/*v*) LEO for 24 h. Representative immunocytochemistry images and respective signal quantification in fixed U87MG cells illustrate the fluorescence intensity of 8-OH(d)G (red) and 4-HNE (red). Nuclei were counterstained with DAPI (blue). Data represent media ± SD. Statistical significance was assessed using the unpaired Student’s *t*-test. ** *p* < 0.01; *** *p* < 0.001.

A plethora of findings highlighted that cancer cells exhibit high basal levels of ROS due to the deregulation of redox metabolism, which results in the generation of oxidized derivatives of biological macromolecules [39]. Immunofluorescence experiments confirmed the presence of oxidized derivatives in our cellular model; in detail, a high expression of both 8-OH(d)G, a marker of oxidative damage to nucleic acids, and 4-HNE, a marker of lipid peroxidation, was observed in U87MG cells. Notably, we observed a strong reduction in both markers in LEO-treated cells, suggesting that LEO can exert antioxidant properties in GBM cells (Figure 4).

### 2.5. Monoterpenes Present in LEO Affect GBM Cell Proliferation

**Figure 5:**
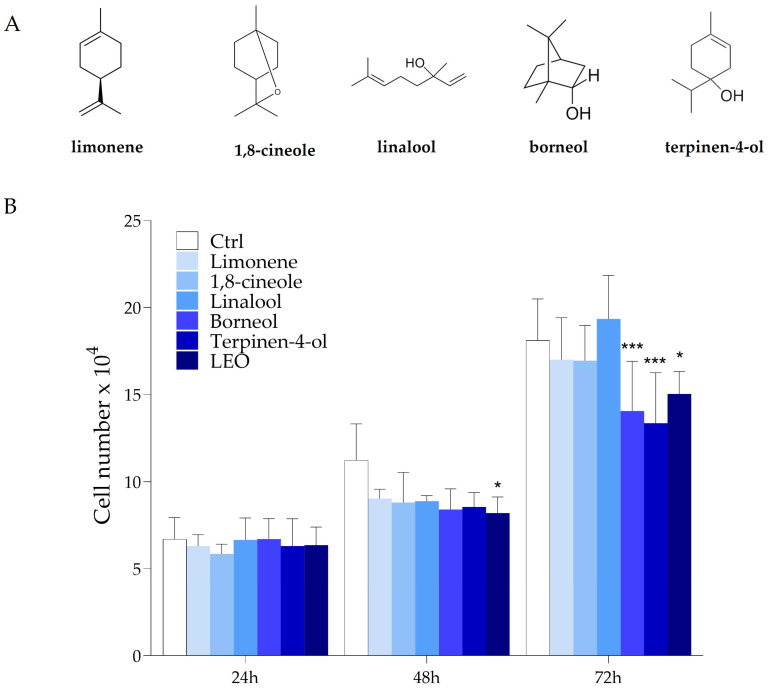
Terpenes enriched in LEO affect cell proliferation. (**A**) Chemical structures of the most abundant terpenes identified in LEO; (**B**) U87MG cells were cultured in DMEM with DMSO as vehicle (Ctrl) or incubated with 1.7 × 10^−3^% (*v*/*v*) linalool, 6.6 × 10^−4%^ (*v*/*v*) borneol, 2.6 × 10^−4^% (*v*/*v*) terpinen-4-ol, 3.1 × 10^−4^% (*v*/*v*) limonene, or 3.1 × 10^−4^% (*v*/*v*) 1,8-cineole for 24, 48, and 72 h. At these time points, the cells were trypsinized and counted, and growth curves were plotted. Data are presented as means ± SD from three independent experiments. Statistical analysis was performed using the two-way ANOVA and Bonferroni’s post hoc test. Asterisk indicates statistical difference vs. Ctrl group at 24, 48, and 72 h. * *p* < 0.05; *** *p* < 0.001.

The essential oil employed in this study is rich in oxygenated monoterpenes, as shown in Table 1, and monoterpenes have been reported to exhibit anticancer properties in several cancer models, including GBM [31,40,41]. To determine whether the observed growth inhibition was due to the monoterpenes and monoterpenoids enriched in LEO, we performed cell proliferation assays on U87MG cells treated with each of the terpenic molecules most represented in LEO. In detail, cells were exposed to each purified compound at the concentration as in a 5 × 10^−3^% (*v*/*v*) LEO solution. Specifically, cells were treated with 1.7 × 10^−3^% *(v*/*v)* linalool, 6.6 × 10^−4^% *(v*/*v*) borneol, 2.6 × 10^−4^% (*v*/*v*) terpinen-4-ol, 3.1 × 10^−4^% *(v*/*v)* 1,8-cineole, and 3.1 × 10^−4^% (*v*/*v*) limonene for up to 72 h. In Figure 5A, the chemical structure of each molecule employed is shown. Figure 5B illustrates that both terpinen-4-ol and borneol markedly reduced the proliferation of U87MG cells after 72 h, to a similar extent as LEO. Although present at the lowest concentration among the monoterpenes analyzed, terpinen-4-ol was able to significantly affect cell proliferation in our model. Therefore, it was selected for subsequent experiments in order to investigate its ability to reproduce the cellular effects obtained with LEO administration.

### 2.6. Terpinen-4-Ol Reproduces the Biological Effects of LEO in U87 Cells

**Figure 6:**
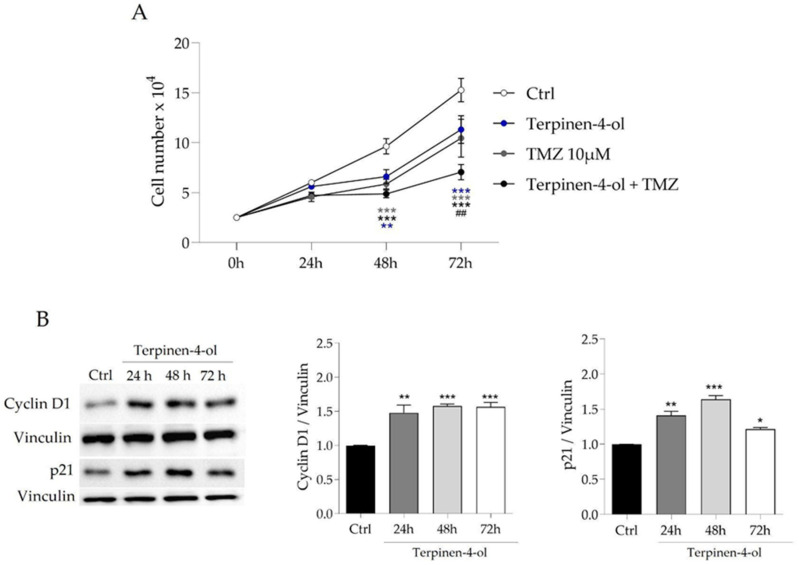
Terpinen-4-ol synergizes with TMZ by upregulating p21 and Cyclin D1. (**A**) U87MG cells were cultured in DMEM with DMSO as vehicle (Ctrl) or treated with 2.6 × 10^−4^% (*v*/*v*) terpinen-4-ol, or 10 μM TMZ and terpinen-4-ol + TMZ for 24, 48, and 72 h. After trypsinization, cells were counted as described and growth curves plotted. Data are shown as means ± SD of three independent experiments. Statistical analysis was performed using the two-way ANOVA and Bonferroni’s post hoc test. Asterisk indicates statistical difference vs. Ctrl group; ## indicates statistical difference vs. TMZ group, *p* < 0.01; ** *p* < 0.01; *** *p* < 0.001. (**B**) Representative western blot and densitometric analysis of p21 and Cyc D1 proteins in U87MG cells cultured in DMEM with DMSO (Ctrl) or treated with 2.6 × 10^−4%^ (*v*/*v*) terpinen-4-ol for 24, 48, and 72 h. Vinculin was used as loading control. Data are presented as means ± SD of three independent experiments. Statistical significance is assessed with one-way ANOVA test, followed by Tukey’s post hoc and indicated vs. Ctrl as follows: * *p* < 0.05; ** *p* < 0.01; *** *p* < 0.001.

We decided to investigate whether terpinen-4-ol was able to reproduce the effects of LEO on GBM cells. First, we evaluated a possible additive effect of terpinen-4-ol and TMZ on cell proliferation. To this end, U87MG cells were incubated with a combination of 2.6 × 10^−4^% (*v*/*v*) terpinen-4-ol and 10 µM TMZ for 24, 48, and 72 h. As shown in Figure 6A, the co-administration significantly enhanced the antiproliferative effect compared to TMZ alone. Indeed, the combination of terpinen-4-ol and TMZ induced a significant reduction in cell proliferation at 72 h, suggesting that this monoterpene enhances the anti-proliferative activity of TMZ. We also analyzed the expression levels of key p21 and cyclinD1 proteins and we found that terpinen-4-ol induces the upregulation of the two cell cycle players, as observed in LEO-treated cells (Figure 6B).

**Figure 7:**
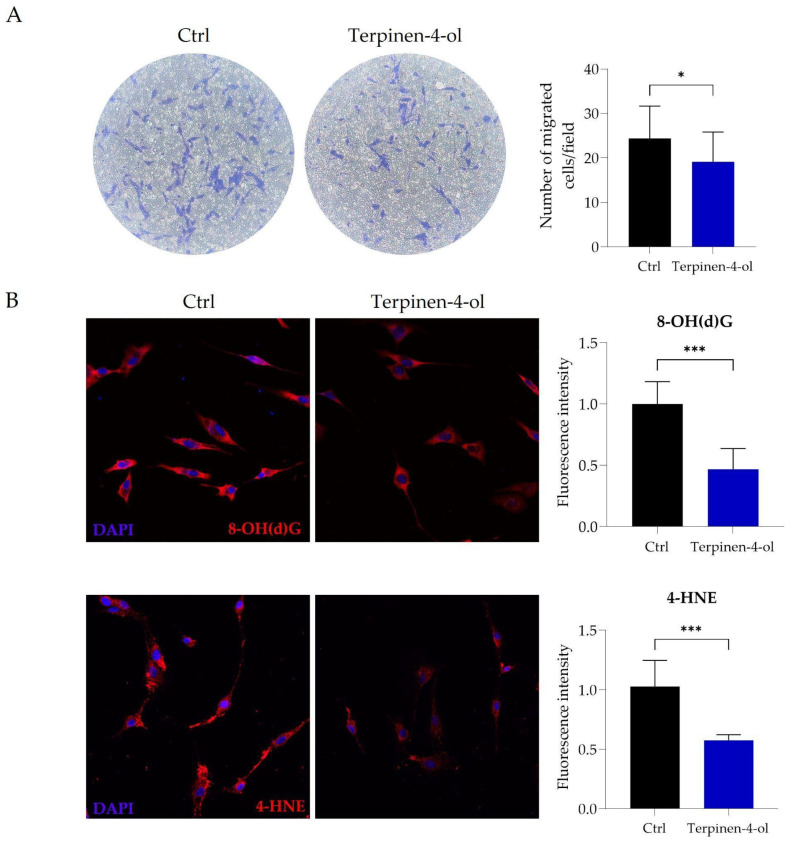
Terpinen-4-ol reproduces the effects of LEO in U87 cells. (**A**) Representative images (20× magnification) of Transwell migration assay of Ctrl and 2.6 × 10^−4^% (*v*/*v*) terpinen-4-ol-treated U87MG cells and quantitative analysis of the number of migrating cells per field obtained through a Transwell migration assay, as previously reported. (**B**) U87MG cells were cultured in DMEM with DMSO (Ctrl) or treated with 2.6 × 10^−4^% (*v*/*v*) terpinen-4-ol for 24 h. Representative immunocytochemistry images and respective signal quantification on U87MG fixed cells of 8-OH(d)G (red) and 4-HNE (red). Nuclei were counterstained with DAPI (blue). Data represent median ± SD. Statistical significance was determined using the unpaired Student’s *t*-test. * *p* < 0.05; *** *p* < 0.001.

Cell migration was then examined, and, as shown in Figure 7A, a reduction in migrated cells was observed after 24 h of treatment using a Transwell assay. Moreover, we observed a marked reduction in the fluorescence intensity of the oxidative stress markers 8-OH(d)G and 4-HNE. This reduction was observed 24 h post-treatment with terpinen-4-ol, analogous to what was previously seen with LEO (Figure 7B). The decreased fluorescence intensity can suggest a reduction in cellular oxidative stress, underscoring the potential of terpinen-4-ol as an antioxidant molecule.

## 3. Discussion

Current standard care protocols for GBM patients typically involve a multimodal approach following surgical resection. This usually includes chemotherapeutic agents such as TMZ in combination with radiotherapy, a regimen commonly known as the Stupp protocol [42,43]. Despite these efforts, the mean survival rate for GBM patients has seen limited improvements over the past decade, with a disheartening 5-year survival rate still below 9.8% [44]. One of the primary challenges in treating GBM is the development of resistance to these therapeutic agents, alongside the significant side effects they induce [45]. Consequently, interest in identifying possible new therapeutic strategies and evaluating the efficacy of new active molecules is growing in the research community. Several preclinical and clinical studies have highlighted the benefits of integrating numerous phytocompounds with conventional anticancer treatments [46,47]. Essential oils are the key constituents of medicinal herbs, and their biological activities have been known since ancient times and are enormously utilized in pharmaceutical industries. It is noteworthy that essential oils possess important biological properties like antibacterial, antioxidant, antiviral, and insecticidal activities [48,49]. The low toxicity and beneficial effects have contributed to their extensive use in promoting both physical and mental well-being. Essential oils have been also proposed as potential anticancer adjuvants due to evidence demonstrating that they may prevent, inhibit, or even reverse the formation of cancerous cells [50,51]. Our research has explored the effects of essential oil extracted from *L. angustifolia* on an in vitro model of GBM. To start, we evaluated whether LEO administration may affect cell proliferation and viability. To this end, the administration of sub-lethal LEO concentrations was employed to exclude non-specific cytotoxicity phenomena [23,52]. We observed that LEO induced a dose- and time-dependent slowdown of cell proliferation, without leading to cell death phenomena or evident morphological alterations. We also found that LEO administration enhanced the antiproliferative effect exerted by the drug TMZ, thus indicating an additive effect of the two molecules. In line with the effect of LEO on slowing down cell proliferation, we observed an accumulation of the cell cycle regulators p21 and cyclin D1. p21 is a cyclin-dependent kinase (CDK) inhibitor (CKI) that effectively suppresses the activity of cyclin–CDK complexes, including CDK1, CDK2, and CDK4/6, thus regulating cell cycle progression, irrespective of cyclin abundance [53,54]. The dysregulation of cell cycle regulators after treatment with essential oils extracted from several plants such as *Citrus limettioides*, *Origanum onites*, and *Rosmarinus officinalis*, among others, has been observed in both in vitro and in vivo studies on non-small-cell lung cancer [55,56]. In addition to affecting cell proliferation, we observed that LEO led to a significant reduction in the migratory capability of GBM cells, suggesting a less aggressive tumor phenotype. Notably, the capacity of GBM cells to disseminate throughout the surrounding parenchyma is a major contributory factor in the tumor’s aggressiveness and is closely associated with poor prognosis [57,58]. Furthermore, due to the well-documented role of essential oils as antioxidant agents, we also investigated the effect of LEO, if any, on the levels of oxidative stress within GBM cells. Indeed, LEO has been demonstrated to enhance glutathione levels and the activity of pivotal antioxidant enzymes including catalase, thereby preventing oxidative stress [59,60]. Similarly to other cancer models, GBM cells display elevated basal levels of ROS compared to normal cells, primarily due to an imbalance between pro-oxidant and antioxidant molecules [61,62], and growing evidence suggests that oxidative stress may represent a crucial aspect in GBM biology, as it may promote cell proliferation and tumor cell survival by activating several oncogenic signaling pathways [61]. Sustained oxidative stress has also been associated with the radio- and chemoresistance of GBM [63]. Our results show that LEO effectively reduces oxidative damage in the cellular environment. Indeed, the decreased levels of 4-HNE, a product of lipid peroxidation capable of chemically modifying proteins, and of 8-OH(d)G, generated following the chemical oxidation of cellular guanosines, suggest a protective role of LEO against oxidative stress that fosters tumor aggressiveness.

In order to discriminate the contribution of the individual molecules contained in the essential oil to the biological effects observed, we decided to incubate GBM cells with LEO-enriched terpenic components. GC-MS analysis performed on Pesche LEO has identified 53 individual components and highlighted the abundance of linalool (33.99%), borneol (13.21%), 1,8-cineole (6.29%), and terpinen-4-ol (5.24%). Terpenes, a heterogeneous group of plant-derived compounds, have been indicated as promising antitumor agents acting at several stages of tumor progression. Indeed, they can suppress early tumorigenesis by inducing cell cycle arrest, inhibiting cell differentiation, and triggering apoptosis in several cancer models [41]. In later stages of cancerogenesis, terpenes may also inhibit angiogenesis and metastasis by modulating key intracellular signaling pathways [27]. We observed that both borneol and terpinen-4-ol were able to reproduce the effects of LEO on GBM cell proliferation. Among the LEO-enriched terpenes that have been demonstrated to be effective in the inhibition of cell proliferation, terpinen-4-ol is particularly relevant due to its notable antitumor properties and ability to induce cell cycle arrest, as evidenced in models of melanoma, lung, colorectal, pancreatic, prostate, and gastric cancers [64]. Additionally, murine xenograft models of lung tumors highlight the antiproliferative effects of terpenes, which induce G0/G1 cell cycle arrest by regulating the expression of Cdk4, cyclin D1, p21, and p27 [65,66,67]. In line with results obtained on GBM cells by Cao et al. [34], we observed that, despite being present at low concentrations among the terpenes analyzed, terpinen-4-ol exhibited a significant antiproliferative activity, alone or in combination with TMZ. We decided to explore the impact of terpinen-4-ol on the migration capability of cells and, as for LEO, we observed, for the first time, a significant impairment of GBM cell migration. Moreover, this terpene effectively reduced oxidative cell damage, providing protection against the increased oxidative stress associated with carcinogenesis. As terpinen-4-ol fully reproduces the effects of LEO on the oncogenic properties of GBM cells, we can speculate that it is, at least partially, responsible for the biological activity exerted by LEO. However, we cannot rule out the possibility that other monoterpenes, such as borneol, could elicit a similar biological effect to that observed with terpinen-4-ol on GBM cells. Our study highlights the potential role of monoterpenes as putative adjuvants in the management of GBM. The combination with traditional drugs could also offer the advantage of mitigating the side effects often associated with high-dose chemotherapy.

## 4. Materials and Methods

### 4.1. Plant Material and Isolation of Essential Oil

*L. angustifolia* flowers were gathered during the balsamic period in August 2022 at the University Garden of Pesche, situated in Molise Region, Italy (41.60003° N, 14.23701° E). The botanical group of the Department of Bioscience and Territory (DiBT) conducted the plant identification, and a voucher specimen was archived in the Herbarium of the DiBT of the University of Molise. The fresh flowers (100 g) were selected, thoroughly cleaned, dried in darkness for one week, and then subjected to hydrodistillation for three hours to extract the essential oil (LEO) according to the standard procedure described in the *European Pharmacopoeia* [68]. The extract was dried over anhydrous sodium sulfate to remove traces of water and then stored in dark vials at 4 °C prior to gas chromatography–mass spectrometry (GC-MS) analysis.

### 4.2. GC-MS Analysis

The analysis of LEO was performed using a Trace GC Ultra gas chromatography system (Thermo Fisher Scientific, Waltham, MA, USA). It was equipped with an Rtx^®^-5 Restek capillary column (Restek, Bellefonte, PA, USA) (30 m × 0.25 mm i.d., 0.25 µm film thickness) coupled with an ion-trap mass spectrometry (MS) detector, specifically the Polaris Q (Thermo Fisher Scientific, Waltham, MA, USA). For injection, a programmed temperature vaporizer (PTV) injector was employed in conjunction with a chromatography station Xcalibur on a PC. The ionization voltage was set at 70 eV, with a source temperature of 250 °C. Full scan acquisition in positive chemical ionization mode ranged from *m*/*z* 40 up to 400 atomic mass units (a.m.u.) at a scan rate of 0.43 scans per second. The column temperature profile started at 40 °C for 5 min, and then increased gradually to 250 °C at a rate of 3 °C/min and was held isothermally for 10 min. Helium gas served as the carrier at a flow rate of 1.0 mL/min. Before injection, each sample (1 µL) was dissolved in n-hexane (1:500 n-hexane solution). The experiment was replicated three times for validation purposes.

### 4.3. Identification of Essential Oil Components

The components were named by comparing their mass spectra fragmentation patterns with those stored in the NIST 02, Adams, and Wiley 275 mass spectral libraries [38,69,70]. Additionally, their retention indices were calculated compared to a series of n-alkane C8–C20. The average relative contents (%) of the sample components were decided from peak areas obtained in triplicate without any adjustments [71,72,73]. All analytical standard components employed (n-alcane C8–C20, linalool, borneol, 1,8-cineole, limonene, terpinen-4-ol, camphor, and lavender oil) were bought from Merck Life Science, Milan, Italy.

### 4.4. Cell Culture and Treatments

The human GBM U87MG cell line was kindly provided by Prof. G. Velasco from Complutense University, Madrid, Spain. The U87 cells were cultured at 37 °C in Dulbecco’s Modified Eagle’s Medium (DMEM) with high glucose, supplemented with 10% fetal bovine serum (FBS), 2 mM L-glutamine, and penicillin/streptomycin solution, and supported at 5% CO_2_. Eight hours after seeding, the U87 cells were treated with 5 × 10^−4^, 5 × 10^−3^, and 5 × 10^−2^% in DMEM (*v*/*v*) of LEO. Based on the chemical–analytical characterization of the oil and the abundance of each compound, individual terpenes were tested at the same concentration found in LEO, specifically 1.7 × 10^−3^% *(v*/*v*) linalool (74856, Merck Life Science, Milan, Italy); 6.6 × 10^−4^% (*v*/*v*) borneol (420247, Merck Life Science, Milan, Italy); 2.6 × 10^−4^% *(v*/*v*) terpinen-4-ol (86477, Merck Life Science, Milan, Italy); 3.1 × 10^−4^% (*v*/*v*) limonene (86477, Merck Life Science, Milan, Italy); and 3.1 × 10^−4^% (*v*/*v*) 1,8-cineole (0002-05-90, HWI pharma services GmbH, Rülzheim, Germany). To facilitate solubilization in the growth medium, the oil and individual compounds were first dissolved in FBS (at a final concentration of 10% in DMEM) before being added to the DMEM. Cells treated with the vehicle (DMSO dilution 1:1000 in cell culture medium) served as the control. For the proliferation assays, 10 µM of Temozolomide (Merck KGaA, Darmstadt, Germany) was used for the time indicated.

### 4.5. Proliferation Assays

GBM cells were plated at a density of 30 × 10^3^ cells per well in 24-well plates filled with DMEM supplemented with 10% FBS and then incubated at 37 °C in a 5% CO_2_ environment. After six hours, the cells were subjected to treatment with the different concentrations of LEO, TMZ as described above, or DMSO (as a vehicle). Cell proliferation was assessed by cell counting using a Blutzählkammer THOMA chamber (Merck Life Science, Milan, Italy) at specific time intervals (0, 24, 48, and 72 h) after trypsinization. Each experiment involved a minimum of three replicates for each condition.

### 4.6. Transwell Migration Assay

U87MG cells were detached using trypsin, then pre-incubated, in suspension, with 5 × 10^−3^% (*v*/*v*) LEO, 2.6 × 10^−4^% (*v*/*v*) terpinen-4-ol or DMSO in invasion medium (DMEM without glutamine supplemented with 100 IU/mL penicillin/streptomycin and 25 mM HEPES, pH 7.4) for 1 h at 37 °C. Subsequently, they were plated at a density of 14 × 10^3^ cells per cm^3^ onto Transwell inserts with an 8 µm pore size. A chemotactic FBS gradient was set up between the lower chamber (10% FBS) and the upper chamber (without FBS), where the cells were seeded. Following 24 h of incubation at 37 °C, the cells were fixed with ice-cold 10% trichloroacetic acid (TCA) for 10 min. Cells adhering to the upper side of the filter were removed by scraping, while those migrated through the insert were stained using a solution of 50% isopropanol, 1% formic acid, and 0.5% brilliant blue R 250 (*v*/*v*). Finally, the U87MG cells were counted in more than 20 fields under a light microscope (Eclipse 7s100; Nikon Europe, Amstelveen, The Netherlands) at a 20× magnification.

### 4.7. Cell Lysis and Western Blotting

The protein extracts were prepared by lysing cells with the proper amount of RIPA buffer (50 Mm Tris HCl, pH 7.4; Triton 1%; Na Deoxycholate 0.25%; SDS 0.1%; 150 mM NaCl; 1 mM EDTA; and 5 mM MgCl_2_) supplemented with a protease inhibitor cocktail. After incubation on ice for 20 min, the samples were centrifuged at 12,000 rpm for 15 min at 4 °C. The supernatants were recovered, and the protein concentrations were determined using a Lowry protein assay (Bio-Rad Laboratories, Milan, Italy). Laemmli buffer 5X (Tris-HCl 315 mM, pH 6.8; 2.5% β-mercaptoethanol; 50% glycerol; 10% sodium dodecyl sulfate; and 0.5% Bromophenol Blue) was added to the supernatants and the samples were boiled at 95 °C for 5 min. The protein extracts were separated on SDS-PAGE and then electroblotted onto nitrocellulose (GE Healthcare, Life Sciences, Little Chalfont, Buckinghamshire, UK) using a turbo Trans-blot Transfer system (Biorad Laboratories, Milan, Italy). After blocking with 5% fat-free milk powder in Tris-buffered saline and 0.1% Tween-20, the membranes were probed overnight at 4 °C with primary antibodies: anti-p21 (Santa Cruz Biotechnology, Dallas, TX, USA, sc-6246, dilution 1:500); anti-Cyclin D1 (Santa Cruz Biotechnology, Dallas, TX, USA, sc-954, dilution 1:500); and anti-Vinculin (Santa Cruz Biotechnology, Dallas, TX, USA, sc-73614, dilution 1:5000). Detection was obtained using horseradish peroxidase-conjugated secondary antibody (Bio-Rad Laboratories, Milan, Italy) and the protein antibody immune complexes were visualized with an ECL plus system (GE Healthcare, Life Sciences, Little Chalfont, Buckinghamshire, UK). The respective chemiluminescence signals were recorded using a ChemiDoc MP system (Bio-Rad Laboratories, Milan, Italy). Densitometric analysis was performed using Image J software (version 1.53) for Windows (National Institutes of Health, Bethesda, MD, USA).

### 4.8. Immunocytochemistry and Confocal Analysis

U87 cells were seeded on coverslips and grown in DMEM high glucose with 10% FBS. Cells were treated with 5 × 10^−3^% *(v*/*v*) LEO, 2.6 × 10^−4^% (*v*/*v)* terpinen-4-ol, or DMSO for 24 h. Subsequently, the cells were fixed with paraformaldehyde (4% solution) for 10 min followed by permeabilization with 0.1% Triton X-100 in PBS for 5 min at room temperature, and then blocked in 3% Bovine Serum Albumin (BSA) dissolved in 0.1% PBS Triton for 1 h. For 8-OHdG staining, immunofluorescence was performed with one added step of incubation with 2M HCl for 20 min at room temperature. The 8-OHdG (Santa Cruz Biotechnology, Dallas, TX, USA, sc-66036; dilution 1:100) and 4-HNE (Thermo Fisher Scientific, MA5-27570; dilution 1:100) primary antibodies was incubated overnight at 4 °C and visualized by Alexa 555 Fluor secondary antibodies (ThermoFisher Scientific, Waltham, MA, USA). After nuclear staining with DAPI (D9542, Merck Life Science, Milan, Italy), the coverslips were mounted with Fluoroshield mounting medium (F6182, Merck Life Science, Milan, Italy) and examined under a confocal microscope (TCS SP8; Leica, Wetzlar, Germany). Images were captured using Leica TCS SP8 equipped with a 40× magnification and Leica LAS X Software (version 3.5.5) (Leica Camera, Wetzlar, Germany) for Windows 10.

### 4.9. Statistical Analysis

Each experiment was conducted a minimum of three times. Statistical analysis was performed using GraphPad Prism software, version 5.03 (GraphPad, La Jolla, CA, USA). The results are presented as means ± standard deviations (SDs). An unpaired Student’s *t*-test was performed to compare the means between two experimental groups. For three-group comparisons, statistical significance was assessed using either a one-way analysis of variance (ANOVA) test followed by Tukey’s post hoc test, or a two-way ANOVA followed by Bonferroni’s post hoc test, as specified.

## 5. Conclusions

We have demonstrated that both LEO and its terpenic components could represent promising molecules in addressing the aggressive nature of GBM, with the potential to enhance the effectiveness of TMZ therapy. These natural compounds target key pathological properties of GBM cells, such as proliferation, migration, and oxidative stress, which promote tumor growth and metastasis. The ability of many terpenes to penetrate the blood–brain barrier makes them valuable candidates for GBM treatment, potentially allowing for lower, less toxic chemotherapy doses. By mitigating oxidative damage and protecting healthy cells, LEO and terpinen-4-ol may offer a comprehensive therapeutic approach combining natural and conventional therapies. This strategy could lead to more effective treatments, improved clinical outcomes, and better survival rates for GBM patients.

## Figures and Tables

**Table 1:** The main chemical compounds of LEO are listed in descending order by area percentage > 1%. These components were matched to their respective peaks (N) in the chromatogram in Figure 1.

N	Compounds	Exp RI	Ref RI	Area % ± SD	Abbr.
19	Linalool	1107	1096	33.99± 0.23	AMO
22	Borneol	1171	1169	13.21 ± 0.10	BMO
13	1,8-cineole	1032	1031	6.29 ± 0.31	BMO
12	Limonene	1029	1029	6.12 ± 0.10	MM
24	Terpinen-4-ol	1181	1177	5.24 ± 0.06	MMO
31	Linalyl acetate	1262	1257	5.04 ± 0.07	AMO
21	Camphor	1148	1146	4.36 ± 0.09	BMO
45	(E)-β Farnesene	1460	1456	4.12 ± 0.17	AS
14	cis-ocimene	1042	1037	3.59 ± 0.45	AM
33	Lavandulyl acetate	1295	1290	1.72 ± 0.03	AMO
53	α-bisabolol	1688	1685	1.64 ± 0.20	MSO
15	trans-ocimene	1052	1050	1.21 ± 0.01	AM
20	allo-Ocimene	1133	1132	1.10 ± 0.54	AM
23	Lavandulol	1173	1169	1.10 ± 0.04	AMO

Abbreviations: AM: aliphatic monoterpenes; MM: monocyclic monoterpenes; AMO: aliphatic monoterpenoids; MMO: monocyclic monoterpenoids; BMO: bi- and tricyclic monoterpenoids; AS: aliphatic sesquiterpenes; MSO: monocyclic sesquiterpenoids.

## Data Availability

Data are available within the article.

## Supplementary Materials

The following supporting information can be downloaded at https://www.mdpi.com/article/10.3390/molecules29246044/s1: Table S1: LEO chemical composition reported in GC-MS elution order; Table S2: The components of LEO organized into chemical groups.
