# Supplementary material for: Lavender Essential Oil and Its Terpenic Components Negatively Affect Tumor Properties in a Cell Model of Glioblastoma
Source: Molecules. 2024 Dec 22;29(24):6044. doi: 10.3390/molecules29246044 (PMC11676467; doi:10.3390/molecules29246044)
Supplement: Supplementary file 1 [file molecules-29-06044-s001.zip › molecules-3343884-supplementary.pdf]

Supplementary material of

Lavender Essential Oil and Its Terpenic Components Negatively Affect Tumor Properties in a Cell Model of Glioblastoma

Miriam Russo [1], Noemi Martella [1], Deborah Gargano [1], Francesca Fantasma [1], Chiara Marcovecchio [1], Veronica Russo [2], Maria Antonietta Oliva [2], Marco Segatto [1], Gabriella Saviano [1,*], Sabrina Di Bartolomeo [1,*] and Antonietta Arcella [2]

[1] Department of Biosciences and Territory, University of Molise, 86090 Pesche, Italy; m.russo15@studenti.unimol.it (M.R.); n.martella@studenti.unimol.it (N.M.); d.gargano@studenti.unimol.it (D.G.); fantasma@unimol.it (F.F.); c.marcovecchio1@studenti.unimol.it (C.M.); marco.segatto@unimol.it (M.S.)
[2] IRCCS Istituto Neurologico Mediterraneo NEUROMED, Via Atinense 18, 86077 Pozzilli, Italy; veronica.2306@hotmail.it (V.R.); mariaantonietta.oliva@neuromed.it (M.A.O.); arcella@neuromed.it (A.A.)
* Correspondence: saviano@unimol.it (G.S.); sabrina.dibartolomeo@unimol.it (S.D.B.)

Table S1. LEO chemical composition reported in GC-MS elution order.

N	Compounds	Exp RI	Ref RI	Area % ± SD	Abbr.
1	α-Thujene	927	930	0.12 ± 0.00	BM
2	α-Pinene	933	939	0.60 ± 0.03	BM
3	Camphene	948	954	0.44 ± 0.02	BM
4	Sabinene	974	975	0.01 ± 0.01	BM
5	β-Pinene	976	979	0.24 ± 0.01	BM
6	1-Octen-3-ol	983	979	0.13 ± 0.01	OT
7	Myrcene	992	990	0.78 ± 0.02	AM
8	α-Phellandrene	1000	1002	0.11 ± 0.00	MM
9	3-Carene	1007	1011	0.28 ± 0.01	BM
10	α-Terpinen	1015	1017	0.05 ± 0.00	MM
11	*p*-Cymene	1024	1024	0.11 ± 0.00	MM
12	Limonene	1029	1029	6.12 ± 0.10	MM
13	1,8-Cineole	1032	1031	6.29 ± 0.31	BMO
14	*cis*-Ocimene	1042	1037	3.59 ± 0.45	AM
15	*trans*-Ocimene	1052	1050	1.21 ± 0.01	AM
16	γ-Terpinene	1061	1059	0.21 ± 0.01	MM
17	*cis*-Sabinene Hydrate	1070	1070	0.28 ± 0.01	BMO
18	Terpinolene	1089	1088	0.52 ± 0.01	MM
19	Linalool	1107	1096	33.99 ± 0.23	AMO
20	*allo*-Ocimene	1133	1132	1.10 ± 0.54	AM
21	Camphor	1148	1146	4.36 ± 0.09	BMO
22	Borneol	1171	1169	13.21 ± 0.10	BMO
23	Lavandulol	1173	1169	1.10 ± 0.04	AMO
24	Terpinen-4-ol	1181	1177	5.24 ± 0.06	MMO
25	Cryptone	1188	1185	0.59 ± 0.01	MMO
26	α-Terpineol	1189	1188	0.59 ± 0.01	MMO
27	Exil butanoate	1193	1192	0.26 ± 0.01	OT
28	Isobornyl formate	1230	1239	0.28 ± 0.01	OT
29	Cumin aldehyde	1243	1241	0.33 ± 0.03	MMO
30	Hexyl isovalerate	1247	1244	0.12 ± 0.01	OT
31	Linalyl acetate	1262	1257	5.04 ± 0.07	AMO
32	Bornyl acetate	1288	1288	0.04 ± 0.01	BMO
33	Lavandulyl acetate	1295	1290	1.72 ± 0.03	AMO
34	Hexyl tiglate	1334	1332	0.08 ± 0.00	OT
35	Neryl acetate	1369	1361	0.08 ± 0.01	AMO
36	*trans*-Myrtanol acetate	1387	1386	0.11 ± 0.01	MMO
37	Hexyl hexanoate	1389	1383	0.08 ± 0.01	OT
38	Sesquithujene	1391	1391	0.16 ± 0.00	BS
39	*7-epi*-Sesquithujene	1405	1405	0.11 ± 0.01	BS
40	Longifolene	1408	1407	0.03 ± 0.01	BS
41	*(E)* Caryophyllene	1419	1419	0.92 ± 0.03	BS
42	Linalool butanoate	1425	1423	0.09 ± 0.01	AMO
43	*trans*-α–Bergamotene	1436	1434	0.12 ± 0.01	MS
44	Aromadendrene	1444	1441	0.06 ± 0.01	BS
45	*(E)*-β-Farnesene	1460	1456	4.12 ± 0.17	AS

46	Linalool isovalerate	1468	1468	0.18 ± 0.01	ASO
47	γ-Muurolene	1482	1479	0.32 ± 0.02	BS
48	*(Z)*-α-Bisabolene	1510	1507	0.20 ± 0.02	MS
49	Lavandulyl isovalerate	1513	1509	0.68 ± 0.04	ASO
50	Caryophyllene oxide	1586	1583	0.10 ± 0.01	BSO
51	α-Muurolol	1644	1646	0.09 ± 0.01	BSO
52	*B*-Bisabolol oxide	1659	1658	0.12 ± 0.02	BSO
53	α-Bisabolol	1688	1685	1.64 ± 0.20	MSO

Abbreviations: AM: aliphatic monoterpenes; MM: monocyclic monoterpenes; BM: bi- and tricyclic monoterpenes; AMO: aliphatic monoterpenoids; MMO: monocyclic monoterpenoids; BMO: bi- and tricyclic monoterpenoids; AS: aliphatic sesquiterpenes; MS: monocyclic sesquiterpenes; BS: bi- and tricyclic sesquiterpenes; ASO: aliphatic sesquiterpenoids; MSO: monocyclic sesquiterpenoids; BSO: bi- and tricyclic sesquiterpenoids, OT: others.

Table S2. The components of LEO organized into chemical groups.

**Chemical group**	**Abbreviation**	**Area %** *L. angustifolia*
Aliphatic monoterpenes	AM	6.68
Monocyclic monoterpenes	MM	7.12
Bi- and Tricyclic monoterpenes	BM	1.69
**Monoterpenes**	**M**	**15.49**
Aliphatic monoterpenoids	AMO	42.02
Monocyclic monoterpenoids	MMO	6.86
Bi- and Tricyclic sesquiterpenes	BMO	24.18
**Monoterpenoids**	**MO**	**73.06**
Aliphatic sesquiterpenes	AS	4.12
Monocyclic sesquiterpenes	MS	0.32
Bi- and Tricyclic sesquiterpenes	BS	1.60
**Sesquiterpenes**	**S**	**6.04**
Aliphatic sesquiterpenoids	ASO	0.98
Monocyclic sesquiterpenoids	MSO	1.64
Bi-and Tricyclic sesquiterpenoids	BSO	0.19
**Sesquiterpenoids**	**SO**	**2.81**
**Others**	**OT**	**0.95**

Abbreviations: AM: aliphatic monoterpenes; MM: monocyclic monoterpenes; BM: bi- and tricyclic monoterpenes; AMO: aliphatic monoterpenoids; MMO: monocyclic monoterpenoids; BMO: bi- and tricyclic monoterpenoids; AS: aliphatic sesquiterpenes; MS: monocyclic sesquiterpenes; BS: bi- and tricyclic sesquiterpenes; ASO: aliphatic sesquiterpenoids; MSO: monocyclic sesquiterpenoids; BSO: bi- and tricyclic sesquiterpenoids, OT: others.
